# First contribution to the doryctine fauna (Hymenoptera, Braconidae, Doryctinae) of Farasan Archipelago, Saudi Arabia, with new records and the description of a new species

**DOI:** 10.3897/zookeys.977.56314

**Published:** 2020-10-22

**Authors:** Yusuf A. Edmardash, Usama M. Abu El-Ghiet, Ahmed M. Soliman, Zarrag I. A. Al-Fifi, Neveen S. Gadallah

**Affiliations:** 1 Entomology Department, Faculty of Science, Cairo University, Giza, Egypt Cairo University Giza Egypt; 2 Biology Department, Faculty of Science, Jazan University, Saudi Arabia Jazan University Jazan Saudi Arabia; 3 Plant Protection Department, Desert Research Center, Cairo, Egypt Desert Research Center Cairo Egypt; 4 Plant Protection Department, College of Food and Agriculture Sciences, King Saud University, Riyadh, Saudi Arabia King Saud University Riyadh Saudi Arabia; 5 Zoology Department, Faculty of Science (Boys), Al-Azhar University, Nasr City, Cairo, Egypt Al-Azhar University Cairo Egypt

**Keywords:** Afrotropical region, Braconidae, Doryctinae, Doryctini, Hecabolini, Heterospilini, *
Mimodoryctes
*, Rhaconotini

## Abstract

The doryctine wasp species (Hymenoptera: Braconidae) of Farasan Archipelago (Saudi Arabia) are studied here for the first time. Six species are reported, of which *Mimodoryctes arabicus* Edmardash, Gadallah & Soliman is described and illustrated as a new species. *Neoheterospilus* sp. is most probably a new species but further collecting should be done to obtain the female. Four species are new records for Saudi Arabia as well as for the whole Arabian Peninsula: *Dendrosotinus ferrugineus* (Marshall, 1888), *Hecabalodes anthaxiae* Wilkinson, 1929, *Mimodoryctes proprius* Belokobylskij, 2001, and Rhaconotus (Rhaconotus) carinatus Polaszek, 1994. The newly recorded species are re-described and illustrated.

## Introduction

**Figure 1.**
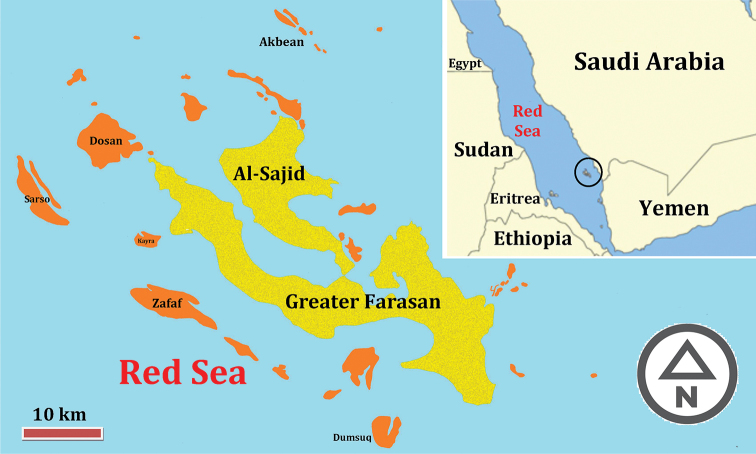
Map of Farasan Archipelago.

The Farasan Archipelago is situated in the southern part of the Red Sea ca. 40 km west of mainland of Jazan mainland coast (Saudi Arabia) [16°41'48"N, 42°7'20"E] (Muoftah 1990; Strumia and Dawah 2019), and has a width of approximately 120 km in SE-NW direction (Alfarhan et al. 2002). A total of 36 big and small islands make up the Farasan group of Islands (Alfarhan et al. 2002), the largest of which is Farasan Al-Kabir (= Greater Farasan, see Fig. 1) (369 km^2^) (Strumia and Dawah 2019). In 1996 Farasan Al-Kabir was established as a protected area by the Saudi Wildlife Commission (SWC), for conserving and restoring animal wildlife, especially the only remaining wild population of Arabian gazelle (El-Demerdash 1996; Alfarhan et al. 2002). Although Farasan lies within the Afro-Asian phytogeographical zone, the floral elements recorded to have the affinity with the Afrotropical, South Palaearctic (Mediterranean) and Oriental regions (Strumia and Dawah 2019). There are no weather stations located in any part of the archipelago, the climate data is therefore is collected from Jazan meteorological station (Alfarhan et al. 2002). The Farasan Archipelago is characterized by the long hot season extending from April to October, and a short mild one (from November to March), with the mean annual temperature is 30 °C, and the mean relative humidity in winter 70–80% and in summer 65–78%.

Among the most important factors that makes Farasan Archipelago unique is the presence of two important Mangrove populations, *Avicennia marina* (Forssk.) (Acanthaceae), and *Rhizophora mucronata* Lam. (Rhizophoraceae), with their ecological and highly productive littoral biotopes which are important as a refuge for many small animals, birds and fish (Mandura et al. 1987). The flora of Farasan comprises 245 species in 152 genera and 52 families (http://ffa.myspecies.info/taxonomy/Term/12). Vegetation along the shoreline of Farasan and Al-Sajid islands is dominated by *Avicennia marina*, whereas Zifaf and Dumsuq islands are dominated by *Rhizophora mucronata* along with *Avicennia marina*. Vegetation in sandy beaches is dominated by halophytes, such as *Aeluropus lagopoides* (L.) (Poaceae), *Cressa cretica* L. (Convolvulaceae), *Halopeplis perfoliata* (Forssk.) (Amaranthaceae), *Limonium axillare* (Forssk.) (Plumbaginaceae), and *Zygophyllum* spp. (Zygophyllaceae) (Alfarhan et al. 2002). Communities of *Vachellia flava* (Forssk.) (Fabaceae), *Blepharis ciliaris* (L.) (Acanthaceae), *Commiphora gileadensis* (L.) (Burseraceae), *Euphorbia fractiflexa* Carter & Wood (Euphorbiaceae), and *Salvadora persica* L. (Salvadoraceae) are also present in almost all the major islands (Alwelaie et al. 1993).

The DoryctinaeFoerster, 1863 is one of the richest, most diverse and most speciose subfamilies of the family Braconidae, second only to Microgastrinae in species richness (Shaw 1995; Marsh 1997; Yu et al. 2016). There are more than 2000 described species in ca. 198 genera and 15 tribes (Braet 2016; Yu et al. 2016; Chen and van Achterberg 2019), and the true number is estimated to be ca. 3000 species. The genus *Heterospilus* Haliday is the most diversified genus in terms of species number and host range (Belokobylskij et al. 2004; Yu et al. 2016). They are mostly distributed in tropical and subtropical regions and are especially diverse in the Neotropical region (Shenefelt and Marsh 1976; Belokobylskij 1992; Marsh 1993, 1997; Marsh et al. 2013). The definition of the subfamily is problematic (Chen and van Achterberg 2019), as is not supported by the use of morphological characters alone, because of the presence of homoplasies (Belokobylskij et al. 2004). It should be revised on the basis of molecular studies (Zaldívar-Riverón et al. 2006, 2008).

Dorytines are cyclostome braconids, diagnosed by the following combination of characters: fore tibia with row or (rarely) cluster of stout pegs along the anterior edge that are distinct from regular setae; hind coxa often with basoventral tubercle; epicnemial and occipital carinae present, which are rarely absent; propleuron with a large, dorso-posterior flange just above the fore coxa, and extending slightly over the ventro-lateral corner of the pronotum; ovipositor strongly sclerotized, distinctly darkened apically; dorsal valve of ovipositor double nodus subapically more or less developed (Quicke et al. 1993; van Achterberg 1993; Marsh 2002). One of the main characters that was traditionally used within doryctine genera is the relative length of basal sternal plate of T1 (= acrosternite *sensu*Belokobylskij 1995). This structure can be short and sessile, or long and petiolate (Belokobylskij 1995; Marsh 1997).

The first attempt to study the evolutionary relationships between the genera of Doryctinae was carried out by Belokobylskij et al. (2004) using morphological characters of 143 genera. However, most of the relationships could not be resolved with the characters used, resulting in an inability to propose a higher classification the subfamily Doryctinae. The monophyly of Doryctinae was also not recovered in some studies, whether based on morphological characters (e.g., Belokobylskij et al. 2004), or on molecular analysis (e.g., Dowton et al. 1998; Zaldivar-Riverón et al. 2007, 2008; Sharanowski et al. 2011), or on a combined morphological and molecular analysis of cyclostome braconids (Zaldivar-Riverón et al. 2006), and so it remains in doubt (Chen and van Achterberg 2019).

Species of the subfamily Doryctinae are not only diverse morphologically but also in their biology (Belokobylskij et al. 2004). From available host records, they are exclusively idiobiont ectoparasitoids of concealed or semi-concealed larvae of wood boring insects, including xylophagous beetles, Lepidoptera and sawflies (van Achterberg 1993; Belokobylskij et al. 2004), termites (Isoptera), and even (as exception) Embioptera (Shaw and Edgerly 1985). A few are known to be phytophagous in seeds (Marsh 1991; de Macêdo and Monteiro 1989; Marsh et al. 2000). Recently, several genera have been discovered to be gall inducers, while others are suspected of being predators of gallers (Zaldívar-Riverón et al. 2007, 2014). In Costa Rica, an unusual biology was discovered in species that are inquilines in figs, where they exhibit an extreme sexual dimorphism that resembles that of chalcid fig wasps (Ramírez and Marsh 1996; van Achterberg and Marsh 2002). A relatively few species are involved in different methods of biological control (Quicke 2015).

No taxonomic studies on this subfamily have been conducted in the Arabian Peninsula. Only three doryctine species have previously been reported there, *Rhaconotus arabicus* Belokobylskij, 2001, *Zombrus anisopus* Marshall, 1897 (Saudi Arabia) (Marshall 1900; Fahringer 1930; Fischer 1980; Belokobylskij 2001), and *Doryctophasmus ferrugineus* (Granger 1949) (United Arab Emirates, Yemen) (Belokobylskij 2015).

## Materials and methods

The present study is based on specimens collected from Farasan Islands (Al-Sajid), using sweeping net and light trap. The specimens including the types of the new species are deposited in the King Saud University Museum of Arthropods, Plant Protection Department, College of Food and Agriculture Sciences, King Saud University, Riyadh, Saudi Arabia (**KSMA**). Genera were identified using Belokobylskij and Tobias (in Tobias et al. 1995), Belokobylskij (2001, 2006), Marsh (2002) and Belokobylskij et al. (2004). On the species level, several available keys, as well as original descriptions were used, like (arranged chronologically): Marshall (1900), Fischer (1968), Papp (1987), Belokobylskij (1983, 1994, 2001, 2006), Polaszek et al. (1994), Belokobylskij and Tobias (in Tobias et al. 1995), van Achterberg and Polaszek (1996), Shaw (1997), van Achterberg and Walker (1998), Shi et al. (2002), Belokobylskij and Maeto (2008), and Tang et al. (2013). The identification of *Rhaconotus carinatus* was confirmed by Andrew Polaszek who kindly examined the holotype (BMNH).

Morphological terminology follows Sharkey and Wharton (1997), Marsh (2002) and Marsh et al. (2013). Wing venation terminology is based on van Achterberg (1993). Body sculpture terminology follows Harris (1979). In the laboratory, the material was studied using a Leica M205 C stereomicroscope. The colour photographs were taken using a Canon EOS 70D camera attached to a Leica MZ 125 stereomicroscope. Individual source images were then stacked using HeliconFocus v.6.22 (HeliconSoft Ltd) extended of field software. Measurements of body parts were made with an ocular micrometer. Further image processing was done using the software Adobe Photoshop CS5.1 (v.12.1 X32) and Adobe Photoshop Lightroom v.5.2 Final (64 bit) [Ching Liu]. The Farasan map (Fig. 1) was plotted from satellite images of Google Earth (accessed 23 October 2019) using ArcGis 10.3, and colored with photoshop Cs6, the scale bar applied only to the magnified map.

Global distribution is based on Yu et al. (2016), in addition to some more recent literature. For tribal classification, we follow Chen and van Achterberg (2019).

### List of abbreviations:

**F** = antennal flagellomeres; **mtn** = metanotum; **ODL** = diameter of ocellus; **OOL** = ocello-ocular line (distance between the outer edge of a lateral ocellus to the compound eye); **POL** = post-ocellar line (distance between the inner edges of the two lateral ocelli); **SOS** = sides of scutellum; **T** = metasomal terga. **Fore wing: 1-R1** = Radial vein; **1-SR+M** = first sector of sectio radii amalgamated with media; **2-SR** = second sector of sectio radii veins; **2-Cu** = second sector of cubital vein; **1**-, **2**- and **3-M** = first, second and third sectors of media, respectively; **3-SR** = third sector of sectio radii veins; **C+Sc+R** = costa, subcosta, and radius amalgamated into one vein; **Hind wing: R1**= radial vein; **SR** = **RS** = sectio radial vein; **SC+R** = subcosta and radius amalgamated into one vein; other veins have the same names as the fore wing.

## Systematic accounts

### Tribe Doryctini Foerster, 1863

#### Genus Dendrosotinus

Taxon classificationAnimaliaHymenopteraBraconidae

Telenga, 1941

E81FD077-6F5E-5927-968B-D37A62199402

Dendrosotinus Telenga, 1941: 80. Type species: Dendrosoter ferrugineus Marshall, 1888, by original designation.

#### Dendrosotinus ferrugineus

Taxon classificationAnimaliaHymenopteraBraconidae

(Marshall, 1888)

56CD5226-021F-5A02-A699-A750F16C8E18

**Figure 2.**
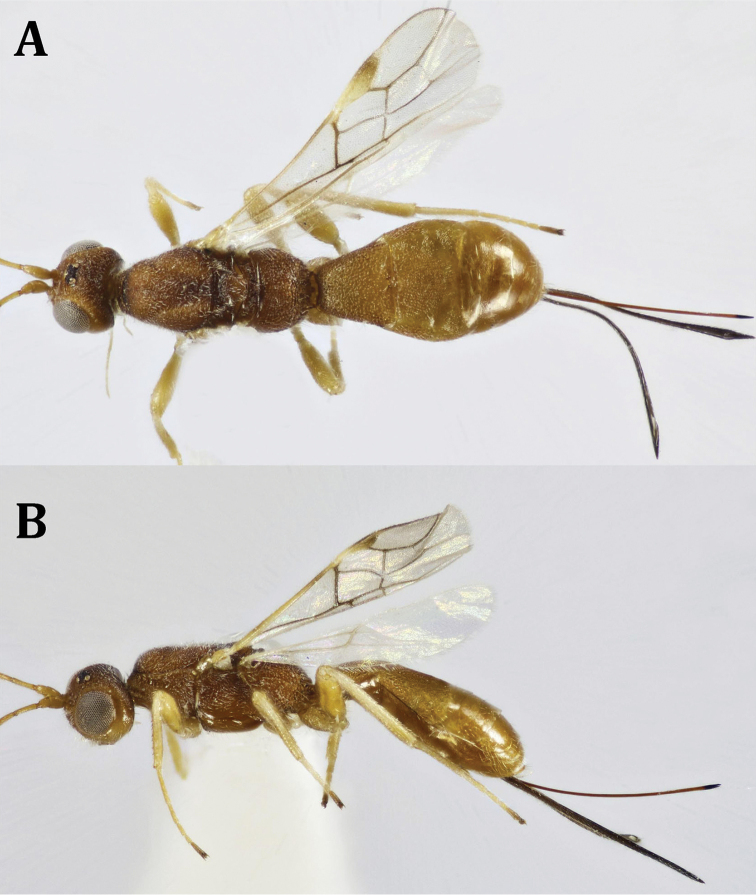
*Dendrosotinus ferrugineus* (Marshall), ♀: **A** dorsal habitus **B** lateral habitus.

**Figure 3.**
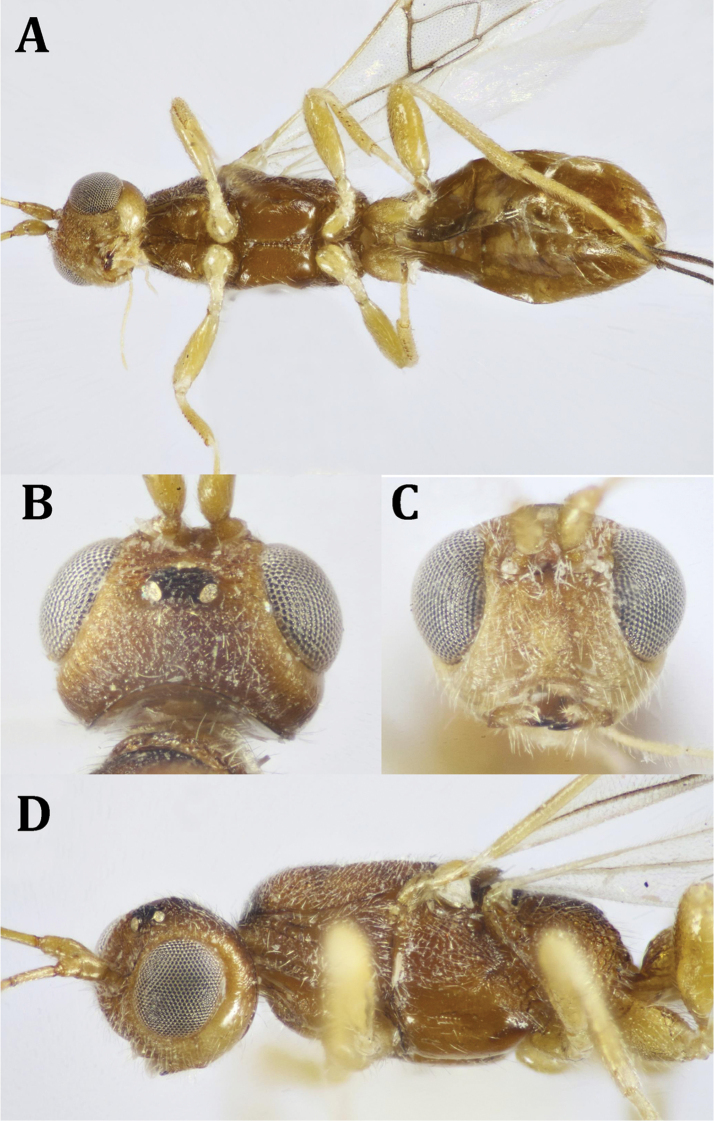
*Dendrosotinus ferrugineus* (Marshall), ♀: **A** ventral habitus **B** head, dorsal view **C** head, frontal view **D** head and mesosoma, lateral view.

**Figure 4.**
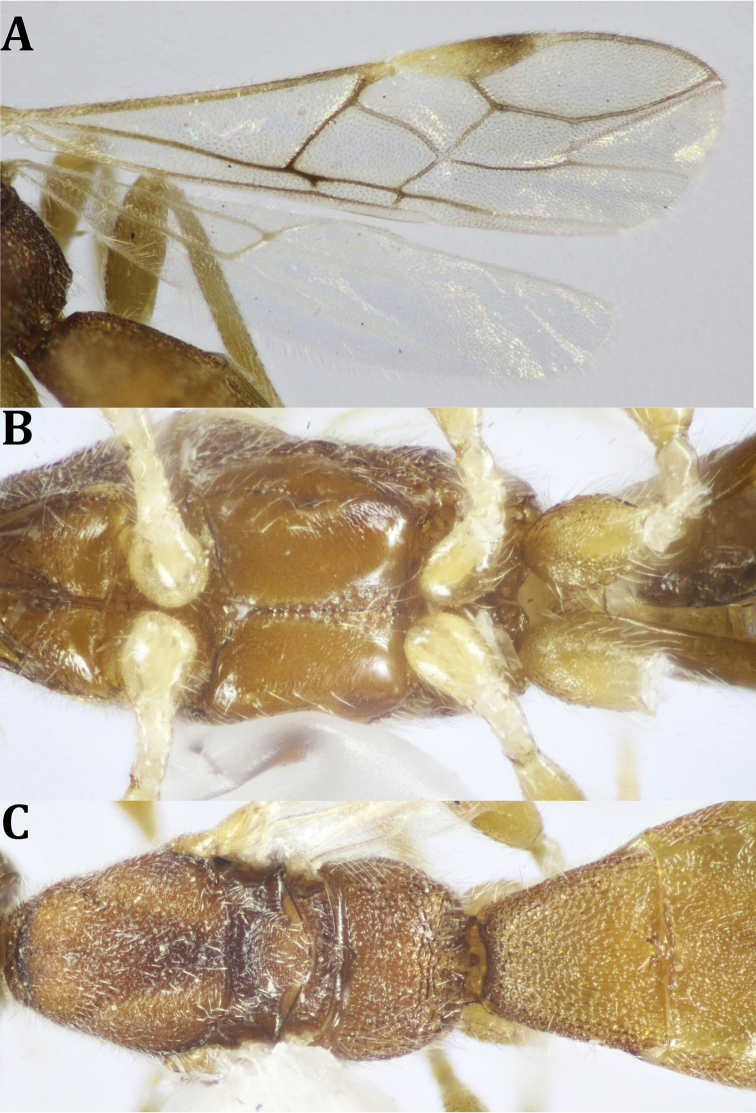
*Dendrosotinus ferrugineus* (Marshall), ♀: **A** fore and hind wings **B** mesosoma, ventral view **C** mesosoma, T1 and T2 (part).

**Figure 5.**
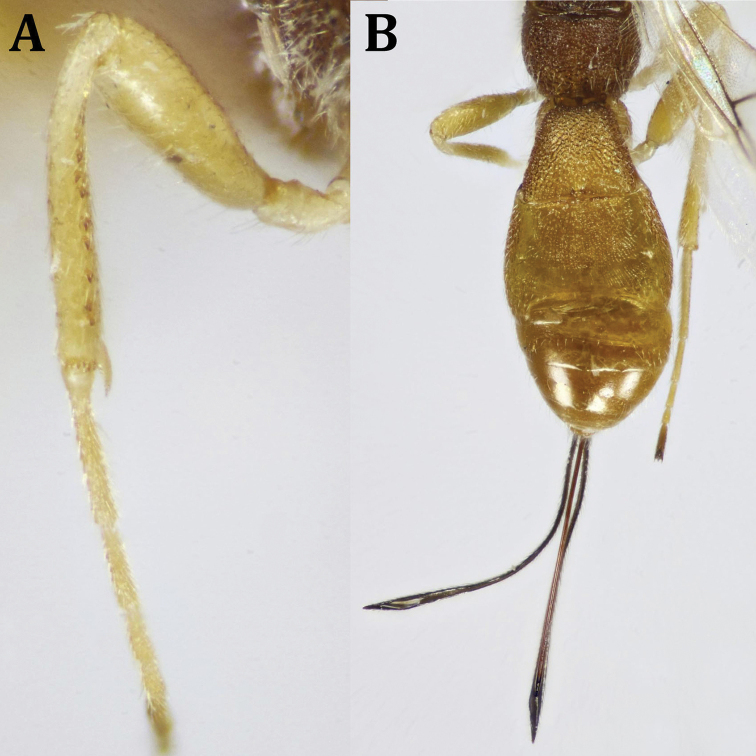
*Dendrosotinus ferrugineus* (Marshall), ♀: **A** hind leg (tibial spines indicated) **B** propodeum and metasoma, dorsal view.

Figures 2A, B , 3A–D , 4A–C , 5A, B

Dendrosoter ferrugineus Marshall, 1888: 247, ♀.

##### Re-description of female.

Body length: 4.8 mm; ovipositor length: 1.4 mm; fore wing length: 2.85 mm.

*Head* (Fig. 3B–D): Slightly wider than mesosoma (1.18×); coarsely rugose dorsally; temple with weak concentric striations, shiny; face coarsely rugose medially, weakly striated laterally behind eyes. Gena rugate above and smooth, with few punctures below. Head constricted behind eyes in dorsal view. Temple 0.58× as long as eye height. POL 1.6× OD, 0.95× OOL. Diameter of antennal socket 2.5× distance between socket to eye edge. Longitudinal eye diameter 1.1× its transverse diameter. Eyes slightly notched opposite to antennal base. Malar space 0.4× eye height, 1.1× as long as basal width of mandible. Face width 0.75× its height including clypeus. Anterior margin of clypeus bended forward, slightly convex; hypoclypeal depression 0.9× distance between depression and eye. Tentorial pits small. Antenna broken (with 10 flagellomeres after being broken); scape short, 1.45× as long as its apical width; F1 5.0× as long as its apical width. Occipital carina thin and sharp, complete dorsally, but not meeting hypostomal carina ventrally.

*Mesosoma* (Fig. 4B, C): 1.9× as long as its maximum height. Pronotum with 6–7 transverse elements. Mesoscutum slightly and gently elevated above pronotum, coarsely rugose, moderately setose. Notauli deep, crenulate; lateral lobes of mesoscutum and anterior end slightly convex. Mesoscutellum about as long as its base, sparsely granulate, with sparse, short whitish setae. SOS smoothly rugate; mtn scrobiculate, with small rounded protrusion postero-medially overlapping base of propodeum, 0.4× as long as mesoscutellum. Propodeum coarsely rugose at basal two-thirds, transversely foveolate at posterior third, with postero-median projections, with long, fine whitish setae laterally and posteriorly. Mesopleuron weakly rugose above, smooth and shiny below; sternaulus short, weakly crenulate, not reaching lateral ends of mesopleuron. Metapleuron strongly areolate.

*Wings* (Fig. 4A): Fore wing with pterostigma 4.3× as long as its maximum width; metacarpe ca. as long as pterostigma. Vein r arising from middle area of pterostigma, 0.5× straight 3-SR, 0.55× 2-SR, 0.75× m-cu; r-m present; discoidal cell 1.9× as long as wide; 3-M entirely unsclerotized; 1-CU1 0.3× as long as 2-CU1, 1-M straight; 1-SR+M slightly curved; M+CU1 straight. Fore wing fringed with short fine setae along its costal and apical margins; hind wing entirely fringed with longer fine setae.

*Legs* (Fig. 5A): Fore femur 2.1× as long as its maximum width; fore and middle tibiae with row of short, thick dark spines along their inner margins; fore tibia with a comb of widely separated short spines distally. Hind tarsus 1.2× as long as hind tibia; hind basitarsus 0.9× as long as remaining hind tarsomeres combined; 2^nd^ tarsomere 0.48× as long as basitarsus, 1.6× as long as telotarsus (excluding arolium).

*Metasoma* (Fig. 5B): Apical width of T1 2.3× as wide as its basal width, 1.3× its median length, densely roughly foveolate; length of T2 + T3 combined 0.7× its basal width, weakly longitudinally striated medially at basal two-thirds, smooth laterally and apically. Remaining tergites smooth and shiny. Ovipositor sheath, ca. as long as metasomal length, 2.88× as long as T1, 1.1× as long as mesosomal length, 0.6× fore wing length.

*Color* (Figs 2A, B, 4A): Head and mesosoma dark brown, metasoma reddish brown, with reddish antenna; palpi pale yellowish, legs yellowish, with dark brown telotarsi. Ovipositor red, with black apex; ovipositor sheath black. Wings hyaline, with pterostigma dark brown, yellow at basal half; parastigma yellowish; all wing veins dark brown. Hind wing with paler veins.

##### Material examined.

Kingdom of Saudi Arabia. 1♀, Jazan, Farasan Islands, Al-Sajid; 16°51'25.46"N, 41°55'58.78"E; 10 Nov. 2017; Usama Abu El-Ghiet & El-Sheikh leg.; LT [KSMA].

##### General distribution.

Armenia, Azerbaijan, Bosnia-Hercegovina, France, Greece, Israel, Italy, Russia, Spain, Turkey, former Yugoslavia (Yu et al. 2016), Saudi Arabia (Farasan Islands) (new record).

### Tribe Hecabolini Foerster, 1863

#### Genus Hecabalodes

Taxon classificationAnimaliaHymenopteraBraconidae

Wilkinson, 1929

826437DC-09AE-5BDA-8392-499B71D7388F

Hecabalodes Wilkinson, 1929: 105. Type species: Hecabalodes anthaxiae Wilkinson, 1929, by original designation.

#### Hecabalodes anthaxiae

Taxon classificationAnimaliaHymenopteraBraconidae

Wilkinson, 1929

765552B3-2E4F-5D30-BD3E-F3589CDA3290

Figures 6A–E , 7A–E

Hecabalodes anthaxiae Wilkinson, 1929: 106, ♀♂.

##### Re-description of female.

**Figure 6.**
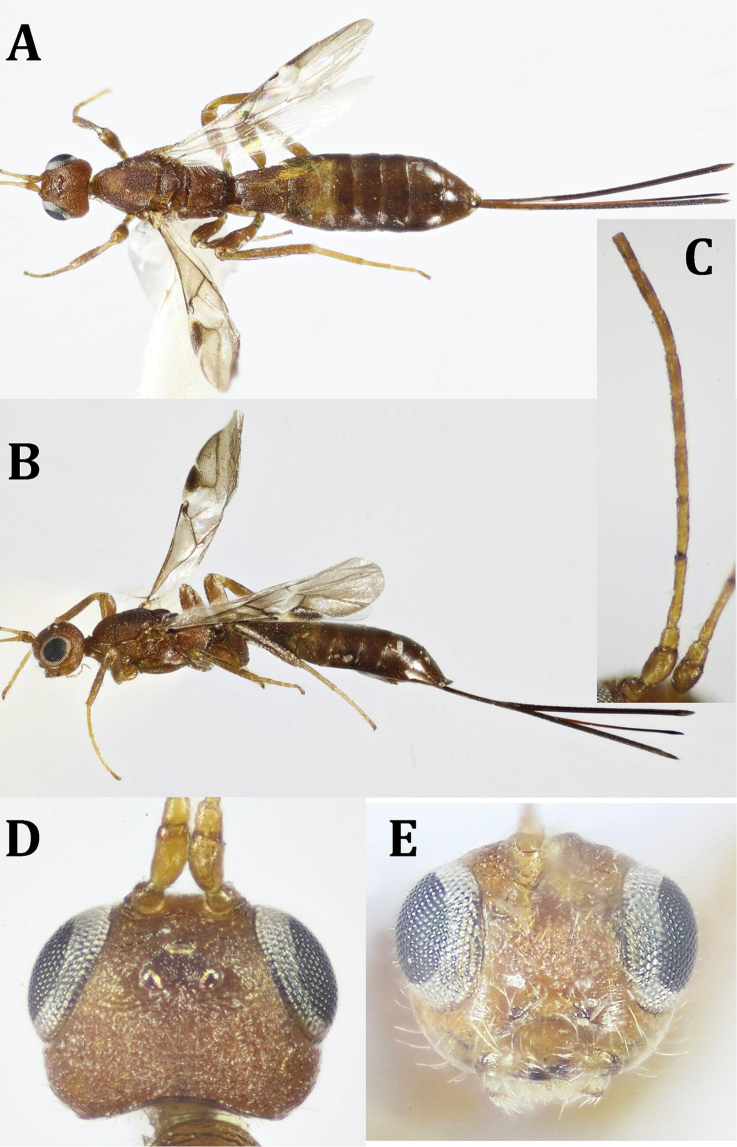
*Hecabalodes anthaxiae* Wilkinson, ♀: **A** dorsal habitus **B** lateral habitus **C** antenna (broken at tip) **D** head, dorsal view **E** head, frontal view.

**Figure 7.**
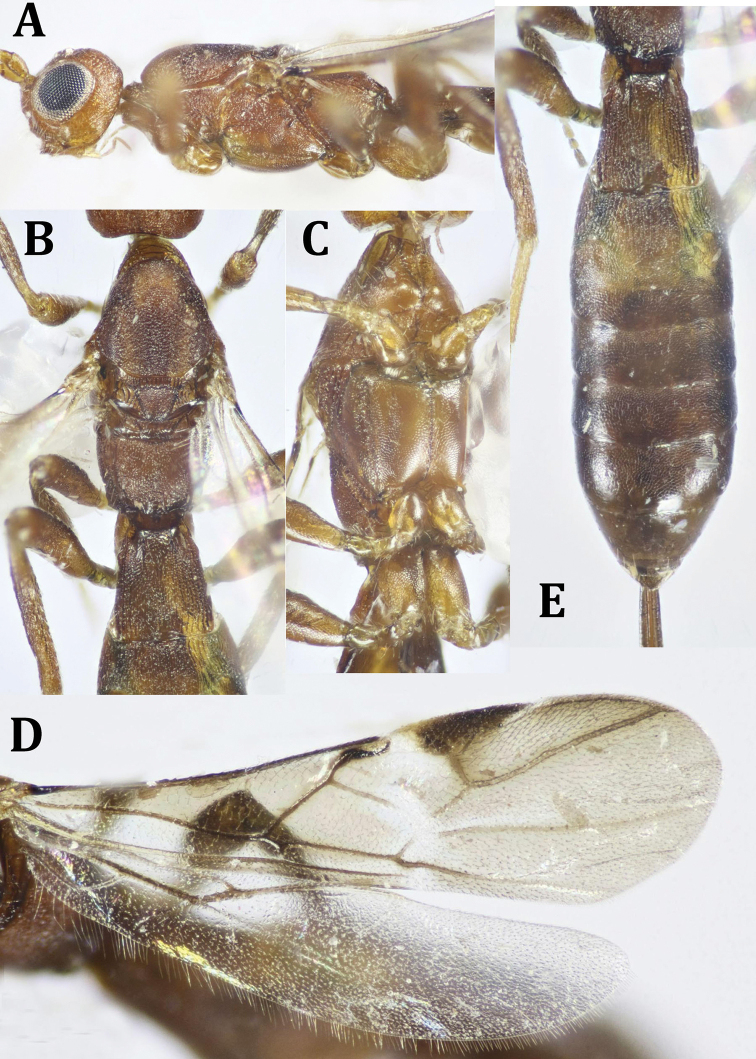
*Hecabalodes anthaxiae* Wilkinson, ♀: **A** head and mesosoma, lateral view **B** mesosoma and metasomal T1 **C** mesosoma, ventral view **D** fore and hind wings **E** metasoma, dorsal view.

Body length: 4.2 mm; ovipositor length: 2.35 mm; fore wing length: 2.5 mm. (we re-describe this species in full because of the short original description of Wilkinson (1929)): Dark brown, except for the yellowish hue on lateral sides of T1 and T2 as well as apex of T2 (Figs 6A, B, 7E); antenna orange, scape slightly darker (Fig. 6C); legs dark brown (except for the yellowish base of fore tibia, and all tarsi), telotarsi darker. Fore wing subhyaline, with distinct infuscation along marginal cell (Fig. 7D).

*Head* (Figs 6C–E, 7A): 1.3× as wide as its median length, slightly wider than mesoscutum; coarsely rugose; head behind eye broadly rounded; temple 0.6× as long as eye height in dorsal view; POL 1.6× OD, 0.9× OOL; eye with few scattered short setae; malar space 0.4× as long as eye height, 1.2× as long as basal width of mandible, malar suture absent; face smooth laterally just behind eyes; face 1.5× as wide as eye width, 0.8× as long its length combined with clypeus; hypoclypeal depression more or less quadrate, ca. as wide as its distance from eye; occipital carina complete dorsally, not meeting hypostomal carina ventrally; antenna broken; scape twice as long as its maximum width; F1 6.5× as long as its apical width, 1.2× as long as F2; ocellar triangle with base longer than lateral sides. *Mesosoma* (Fig. 7A–C): 2.3× as long as its height; pronotum with two sharp transverse carinae dorsally; mesoscutum gently rounded above or at the same level of pronotum when seen from lateral view, flattened on disc, densely rugose, finely alutaceous laterally; notauli indistinct; mesoscutellum slightly convex, truncate at apex, finely sculptured, with a number of thick carinae laterally; mtn 0.4× as long as mesoscutum, with a short longitudinal median carina and 2–3 oblique submedian carinae on its depressed anterior part, convex postero-medially; propodeum finely and sparsely granulate, with two short postero-medial, parallel carinae, 0.3× as long as propodeal length, median longitudinal carina of propodeum absent; mesopleuron finely punctate, with irregular spaces in between, shiny; precoxal sulcus shallow, irregular, running ventrally along almost the entire length of mesopleuron. *Fore wing* (Fig. 7D): 3.6× as long as its maximum width; pterostigma 1.7× as long as maximum width; vein M + CU1 slightly curved; 1-SR+M nearly straight; vein r-m absent; vein r arising at basal third of pterostigma; 2-SR 1.75× as long as r, slightly longer than m-cu, 0.6× as long as 1-SR+M; 1CU1 0.2× as long as 2CU1. *Hind wing* (Fig. 7D): With fringe of long, fine setae along apical and anal margins; vein 1-M 1.7× as long as 1-rm. Legs. Hind coxa (Fig. 7C) 1.7× as long as wide, without distinct basoventral tubercle, finely punctate especially ventrally, with some fine whitish setae distally and laterally; hind femur 2.6× as long as wide; hind tarsus 1.1× as long as hind tibia; hind basitarsus slightly shorter than rest of tarsomeres combined; second tarsomere 0.55× as long as hind basitarsus, 2.2× as long as telotarsus (excluding arolium); outer edge of hind tibia with long, fine whitish setae. *Metasoma* (Figs 6A, B, 7E): 1.3× as long as head and mesosoma combined; T1 and basal half of T2 with distinct interrupted longitudinal striae, somewhat dotted in between; T1 1.5× as long as its apical width; T2 0.9× as long as its apical width, 2.7× as long as T3; posterior half of T2 finely reticulate, T3–5 (except posterior margin of T5 smooth and shiny), finely reticulate; T6 entirely smooth and shiny. Ovipositor sheath about as long as or slightly longer than metasoma (Fig. 6A, B), and the fore wing as well.

##### Material examined.

Kingdom of Saudi Arabia, 1♀, Jazan, Farasan Islands, Al-Sajid; 16°51'25.46"N, 41°55'58.78"E; 10 Nov.2017; Abu El-Ghiet & El-Sheikh leg.; LT [KSMA].

##### General distribution.

Sudan (Wilkinson, 1929), Saudi Arabia (Farasan Islands) (new record).

##### Remark.

This species has not been collected during the 90 years or more since Wilkinson described the holotype from Sudan in 1929.

### Tribe Heterospilini Fischer, 1981

#### Genus Neoheterospilus

Taxon classificationAnimaliaHymenopteraBraconidae

Belokobylskij, 2006

6A9ED0CA-DD81-5A13-BC1D-F954B8E3A8E0

Neoheterospilus Belokobylskij, 2006: 151. Type species: Neoheterospilus koreanus Belokobylskij, 2006, by original designation.

#### Neoheterospilus

Taxon classificationAnimaliaHymenopteraBraconidae

sp.

DB5A188F-54A7-53D1-969E-56ADBC03FD65

Figures 8A–E , 9A–G

##### Description of male.

Body length: 2.25 mm; fore wing length: 1.7 mm.

**Figure 8.**
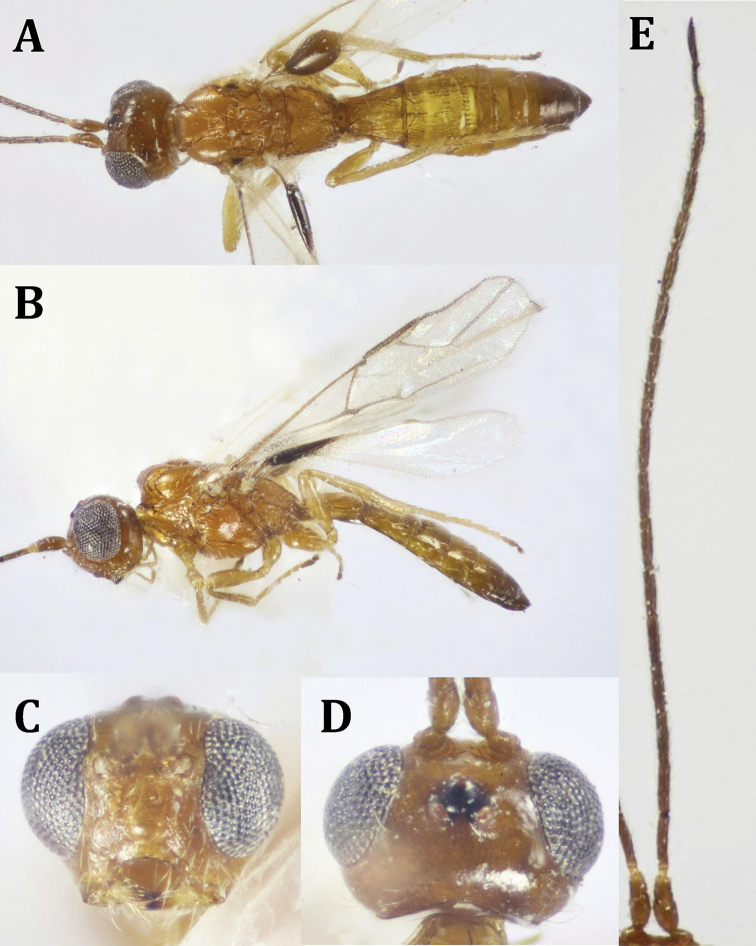
*Neoheterospilus* sp., ♂: **A** dorsal habitus **B** lateral habitus **C** head, frontal view **D** head, dorsal view **E** antenna.

**Figure 9.**
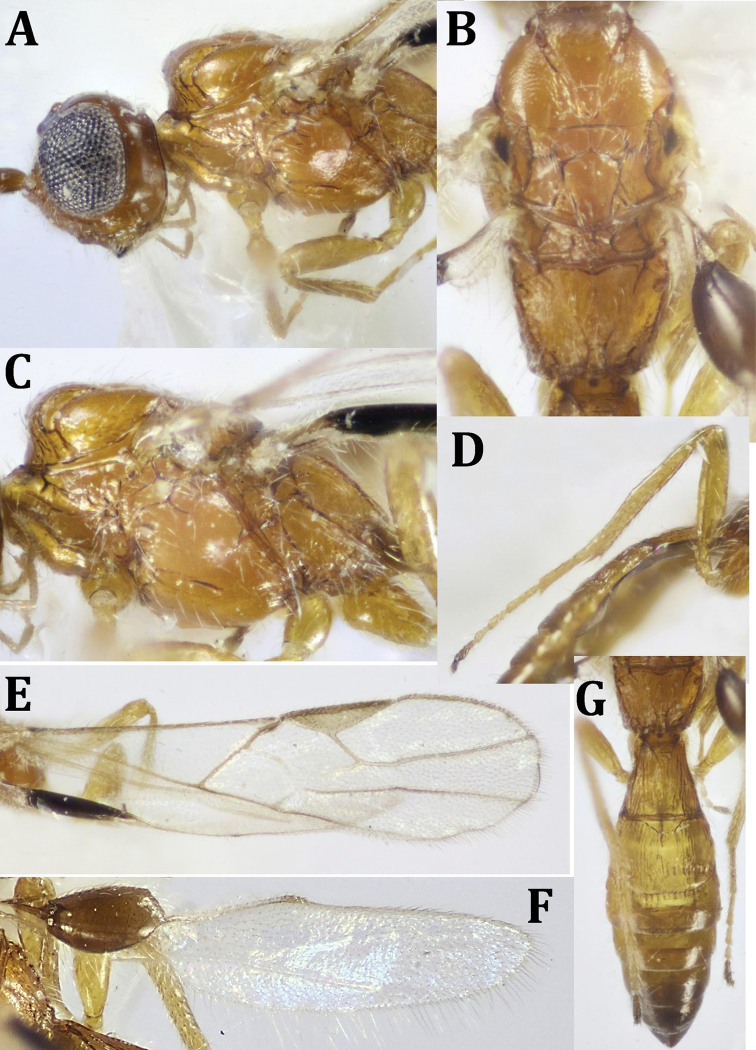
*Neoheterospilus* sp., ♂: **A** head and mesosoma, lateral view **B** mesosoma, dorsal view **C** mesosoma, lateral view **D** hind leg and metasoma (part), lateral view **E** fore wing **F** hind wing **G** propodeum and metasoma, dorsal view.

*Head* (Figs 8C–E, 9A): 0.7× as wide as its median length, distinctly wider than mesoscutum (1.3×). Head below eyes distinctly straight when seen from frontal view. Vertex distinctly smooth and shiny; frons superficially finely punctate, interspaces smooth. Head behind eyes gently rounded when seen from dorsal view; temple smooth, with few scattered setae, 0.6× eye length. Ocelli placed in an equilateral ocellar triangle. POL 1.6× OD, 1.0× OOL; diameter of antennal sockets 1.4× distance between socket and eye. Eye glabrous, slightly emarginate opposite to antennal sockets, 1.1× as high as broad. Malar space 1.1× as long as basal width of mandible, 0.4× as long as eye height; malar suture absent. Face slightly convex, very finely sculptured laterally, nearly smooth medially, with few scattered setae; its width 0.8× height of eye, and 1.2× as wide as its height. Clypeus very thin, transverse, moderately arched at free margin; hypoclypeal depression moderate, semi-oval, its width 0.6× face width. Occipital carina thin, complete dorsally, reaching hypostomal carina ventrally. Antenna slender, filiform, pointed at apex, without spine, 21-segmented, hardly longer than body length; scape nearly smooth, rather short, with few scattered setae, 1.2× as long as wide; flagellum densely setose, F1 slender, straight, 4.9× as long as its apical width, ca. as long as F2; penultimate segment 6.0× as long as F1, 0.7× as long as apical flagellomere.

*Mesosoma* (Fig. 9A–C): Almost smooth, lateral lobes of mesoscutum finely sculptured to alutaceous, not depressed, 1.9× as long as its height. Pronotum rather short, nearly straight, smooth, collar with longitudinal median and lateral carinae. Mesoscutum distinctly high, more or less perpendicularly elevated above pronotum; its maximum width 1.5× as wide as its middle length; median lobe of mesoscutum, slightly, but straightly protruding forwardly. Notauli wide and deep anteriorly, shallow and thinner posteriorly, broad anteriorly and meeting posteriorly before posterior margin of mesoscutum, distinctly foveolate. Prescutellar area in the form of two subquadrate plates, separated medially by a thin linear suture, mostly smooth, 0.4× as long as mesoscutellum. Mesoscutellum slightly convex at anterior half, with very fine lateral carina, its basal width 0.7× its median length. Subalar depression smooth, nearly rounded. Sternaulus moderately deep, straight, smooth, running along median area of lower part of mesopleuron. Metapleural lobe relatively large, nearly smooth, gently rounded posteriorly just above hind coxa. Propodeum smooth, nearly flattened, laterally carinate, with two short, posterior sublateral, oblique and slightly curved carinae at base as well as a median straight one, 0.1× as long as propodeal length; basal sublateral carina could also be seen, 0.4× as long as propodeum length; propodeal spiracle relatively small.

*Wings* (Fig. 9E, F): Fore wing 3.8× as long as its maximum width, 0.75× as long as body length; r arising near to the middle of pterostigma; Radial cell long (not shortened); metacarpus longer than pterostigma; r 1.4× as long as maximum width of pterostigma; 3-SR 0.85× r, forming with it an obtuse angle; 3-SR 0.2× as long as SR1, straight; trace of 1-SR+M distinctly lower than 2-SR+M (very hardly seen to be measured); m-cu slightly curved; brachial cell broadly opened distally. Hind wing 4.6× as long as its maximum width, costal cell absent, Costal vein stigma-like subbasally. Whole edges of both wings surrounded with relatively long fringe of setae.

*Legs* (Fig. 9D): Hind coxa 1.2× as long as its maximum width, with small, but distinct baso-ventral tubercle; hind femur narrow, without blister dorsally, 4.2× as long as its maximum width; hind basitarsus 0.3× as long as hind tibia; hind tibia with weak blister near to the middle, second tarsomere of hind leg 0.64× as long as hind basitarsus.

*Metasoma* (Fig. 8A, B, 9G): Nearly glabrous, except for very few fine long setae laterally, 2.7× as long as its maximum width, 1.1× as long as head and mesosoma combined. T1 widened from base to apex, its apical width 2.1× its basal width, 1.0× its middle length, with small basal dorsope; with baso-median smooth area that narrowed posteriorly, not reaching middle of tergite, with very weak, irregular longitudinal striations that are obscured medially; T1 1.4× as long as propodeal length; T2 with a trace of short, semi-circular smooth area baso-medially; median length of T2 0.8× its basal width, 0.8× as long as T1 and 1.8× as long as T3, sculpturing as in T1, but very superficial and weaker. T3 ca. 2.0× as wide as long, with short, thick, widely separated longitudinal striations at base. Remaining tergites smooth and shiny.

*Color* (Figs 8A, B, 9E, F): Body generally reddish yellow, with head distinctly darker; antenna with scape and pedicel as body color, flagellum dark brown to black; maxillary and labial palpi pale brown; ocellar triangle black, last metasomal tergites dark brown to black. Wings hyaline, fore wing pterostigma and veins dark brown.

##### Material examined.

Kingdom of Saudi Arabia. 1♂, Jazan, Farasan Islands, Al-Sajid; 16°51'25.46"N, 41°55'58.78"E; 25 Jan.2017; Usama Abu El-Ghiet & El-Sheikh leg.; LT [KSMA].

##### Remark.

Although it cannot be matched with any of the species keyed out by Belokobylskij in his paper of *Neoheterospilus* (2006), it should not be described as new until females are collected (Belokobylskij, pers. comm.).

##### General distribution.

Saudi Arabia (Farasan Islands) (new record).

### Tribe Rhaconotini Fahringer, 1928

#### Genus Rhaconotus

Taxon classificationAnimaliaHymenopteraBraconidae

Ruthe, 1854

2BF50701-4848-5B0F-9DCE-BCCBBA40EE93

Rhaconotus Ruthe, 1845: 349. Type species: Rhaconotus aciculatus Ruthe, 1845 (by monotypy)
Hedysomus Foerster, 1863: 238. Type species: Hedysomus elegans Foerster, 1863 (by original designation)
Hormiopterus Giraud, 1869: 478. Type species: Hormiopterus ollivieri Giraud, 1869 (by monotypy)
Euryphrymnus Cameron, 1910: 100. Type species: Euryphrymnus testaceiceps Cameron, 1910 (by monotypy)
Rhaconotinus Hedqvist, 1965: 8. Type species: Rhaconotinus caboverdensis Hedqvist, 1965 (by original description)

#### Rhaconotus (Rhaconotus) carinatus

Taxon classificationAnimaliaHymenopteraBraconidae

Polaszek, 1994

B8EDCEB9-AA6C-5F82-A6B6-0C8E9CE2378D

**Figure 10.**
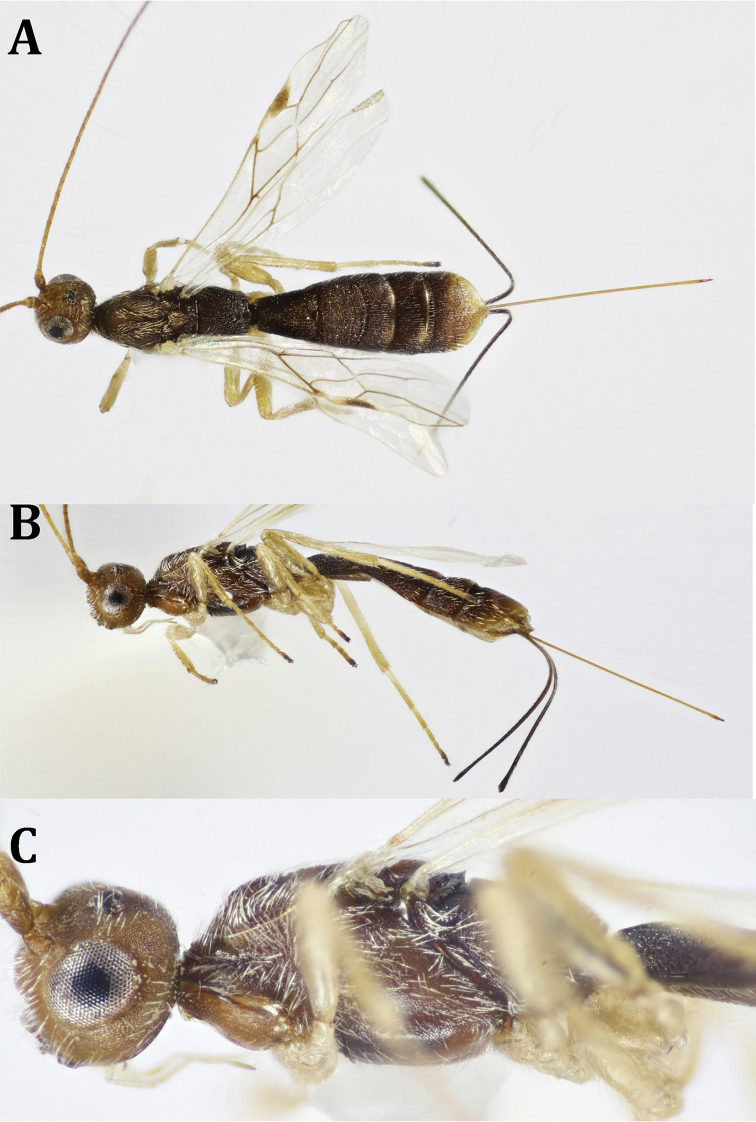
Rhaconotus (Rhaconotus) carinatus Polaszek in Polaszek et al. 1994, ♀: **A** dorsal habitus **B** lateral habitus **C** head and mesosoma, lateral view.

**Figure 11.**
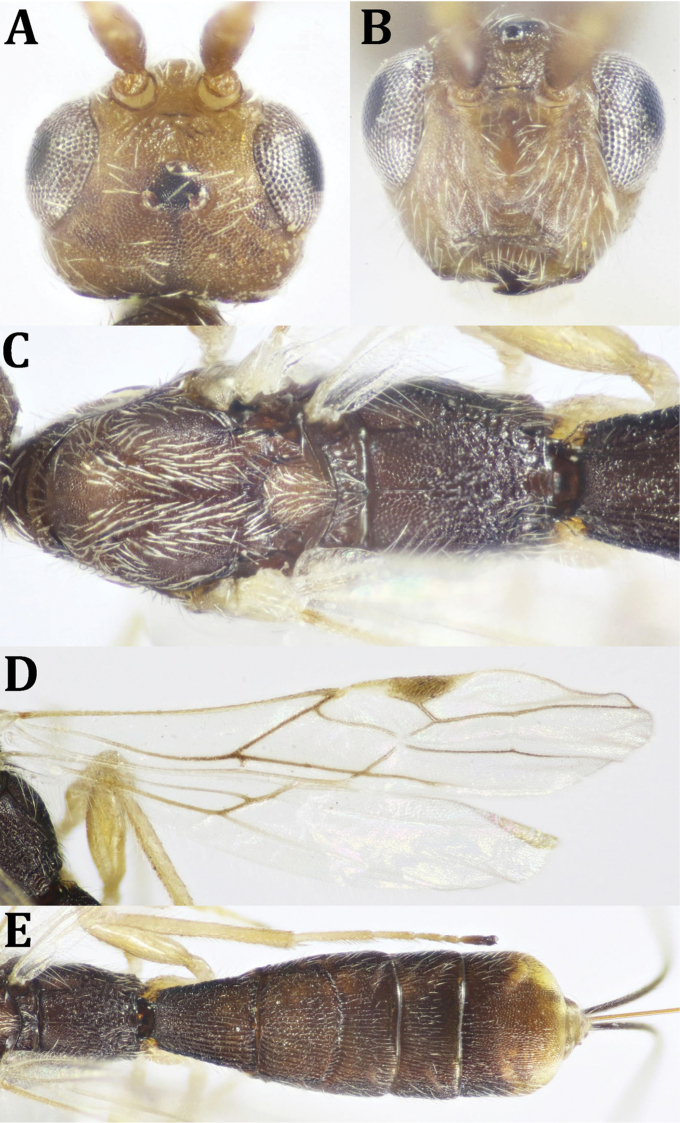
Rhaconotus (Rhaconotus) carinatus Polaszek in Polaszek et al. 1994, ♀: **A** head, dorsal view; **B** head, frontal view **C** mesosoma, dorsal view **D** fore and hind wings **E** propodeum and metasoma, dorsal view.

Figures 10 (A–C), 11 (A–E)

Rhaconotus carinatus Polaszek in Polaszek et al., 1994: 79, ♀.

##### Diagnosis.

Female: Body length: 4.5–4.8 mm; fore wing length: ca. 3.1 mm.

Generally dark reddish brown, with posterior margin of T4 and T5 yellowish in color (Fig. 10A, B) (in some specimens, head reddish, with black ocellar triangle); antenna with scape dark reddish brown, pedicel and basal half of flagellum reddish, rest of flagellum dark brown. Legs and palpi are pale yellowish (except dark brown telotarsus). Wings (Fig. 11D) hyaline, with slight, hardly seen fumigation behind pterostigma; pterostigma brownish, with pale basal and apical ends; veins brownish, with basal three-fourths of C+SC+R, basal two-thirds of 1-R1, and basal half of M+CU1 are pale brownish in color; ovipositor reddish, slightly dark at apex, ovipositor sheath black (Fig. 10A, B).

*Head* (Figs 10C, 11A, B) finely sculptured, with few scattered fine whitish, semi-erect setae when seen from dorsal view; face finely punctate, with distances between punctures, smooth medially just beneath antennal bases, and above hypoclypeal area, with denser appressed setae. Temple 0.6× eye height. Antenna 35-segmented. Mesoscutum (Figs 10C, 11C) with fine reticulation except nearly smooth posteromedially; propodeum finely reticulate, longitudinal median carina hardly seen just at base, as well as two shorter ones baso-laterally. Metasoma (Fig. 11E) with T2 and T3 fused, separated by a strong curved suture or groove, after which the longitudinal striations became weakly visible; T5 simple, broadly rounded posteriorly. Ovipositor sheath ca. as long as metasoma (Fig. 10A, B).

##### Material examined.

Kingdom of Saudi Arabia. 1♀ & 1♂, Jazan, Farasan Islands, Al-Sajid; 16°51'25.46"N, 41°55'58.78"E; 7 Jan.2017; Abu El-Ghiet & El-Sheikh leg.; sweeping net [KSMA]; 1♀, Kingdom of Saudi Arabia, Jazan, Farasan Islands, Al-Sajid; 16°51'25.46"N, 41°55'58.78"E; 10 Nov.2017; Abu El-Ghiet & El-Sheikh leg.; LT [KSMA].

##### General distribution.

Cameroon, Ghana, Madagascar, Nigeria, Senegal, Sierra Leone, Tanzania, Togo (Polaszek et al. 1994), Saudi Arabia (Farasan Islands) (new record).

##### Remark.

Based on Polaszek et al. (1994) and van Achterberg and Polaszek (1996), our species differs from the African specimens in having the pterostigma distinctly infuscate medially, with pale basal and apical ends (distinctly infuscate in the African specimens); antenna 35-segmented (26–33 in the African specimens); lateral lobes of mesoscutum moderately setose (largely glabrous in the African specimens); propodeum finely reticulate, with a hardly visible median longitudinal carinae as well as two very short sublateral ones (almost smooth anteromedially in the African specimens, see fig. 30 in Polaszek et al. (1994) and fig. 366 in van Achterberg and Polaszek (1996)).

### Genera with uncertain tribal relationships

#### Genus Mimodoryctes

Taxon classificationAnimaliaHymenopteraBraconidae

Belokobylskij, 2001

321E2B5B-BE0B-5C0A-9D6A-2CF823BC6191

Mimodoryctes Belokobylskij, 2001: 749.

##### Type species.

Mimodoryctes proprius Belokobylskij, 2001, by monotypy. 

#### Mimodoryctes arabicus

Taxon classificationAnimaliaHymenopteraBraconidae

Edmardash, Gadallah & Soliman sp. nov.

DF6A5C47-B2EA-564B-A1F1-77BB20DC3431

http://zoobank.org/D8226F4F-86DE-4987-A7CD-EABF927009DD

**Figure 12.**
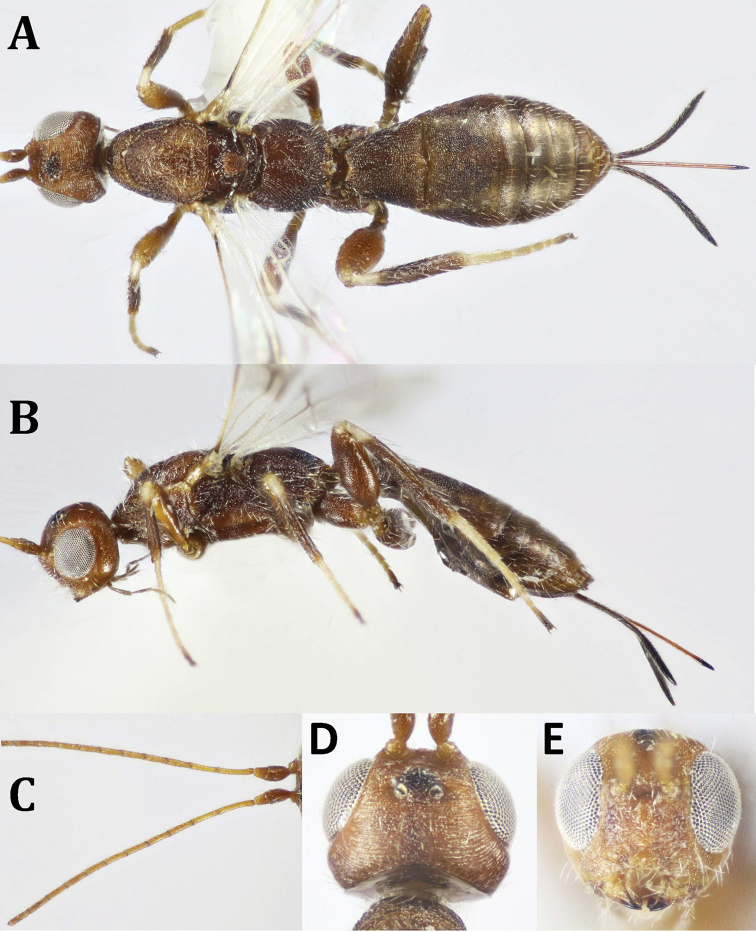
*Mimodoryctes arabicus* Edmardash, Gadallah & Soliman, sp. nov. ♀: **A** dorsal habitus **B** lateral habitus **C** antenna (part) **D** head, dorsal view **E** head, frontal view.

**Figure 13.**
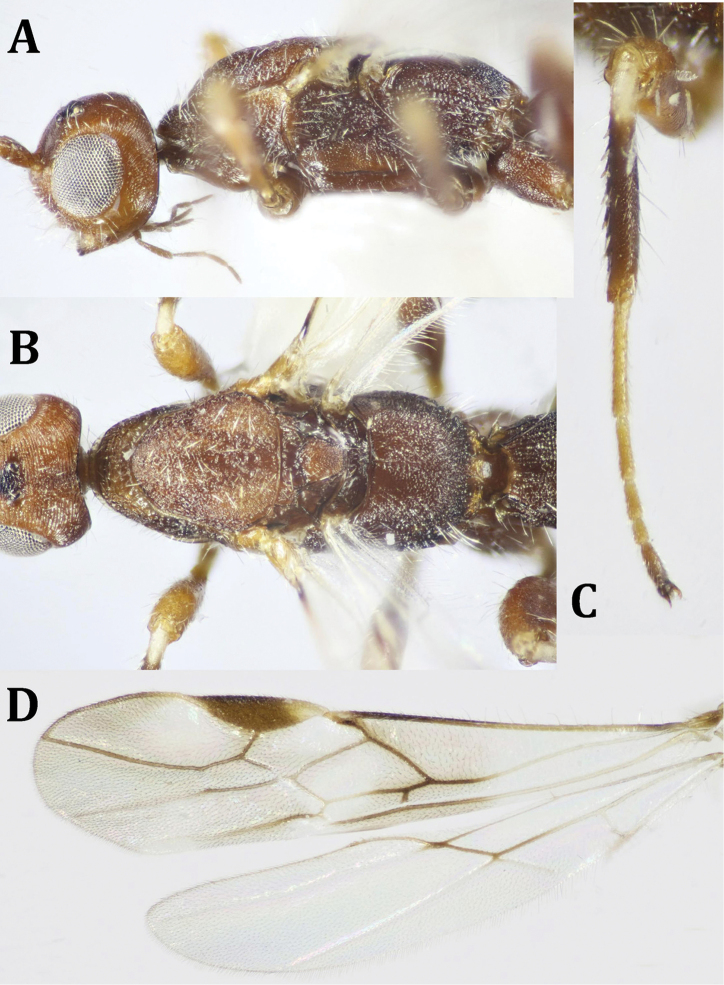
*Mimodoryctes arabicus* Edmardash, Gadallah & Soliman, sp. nov. ♀: **A** head and mesosoma, lateral view **B** head (part) and mesosoma, dorsal view **C** fore leg (fore tibial spines indicated) **D** fore and hind wings.

**Figure 14.**
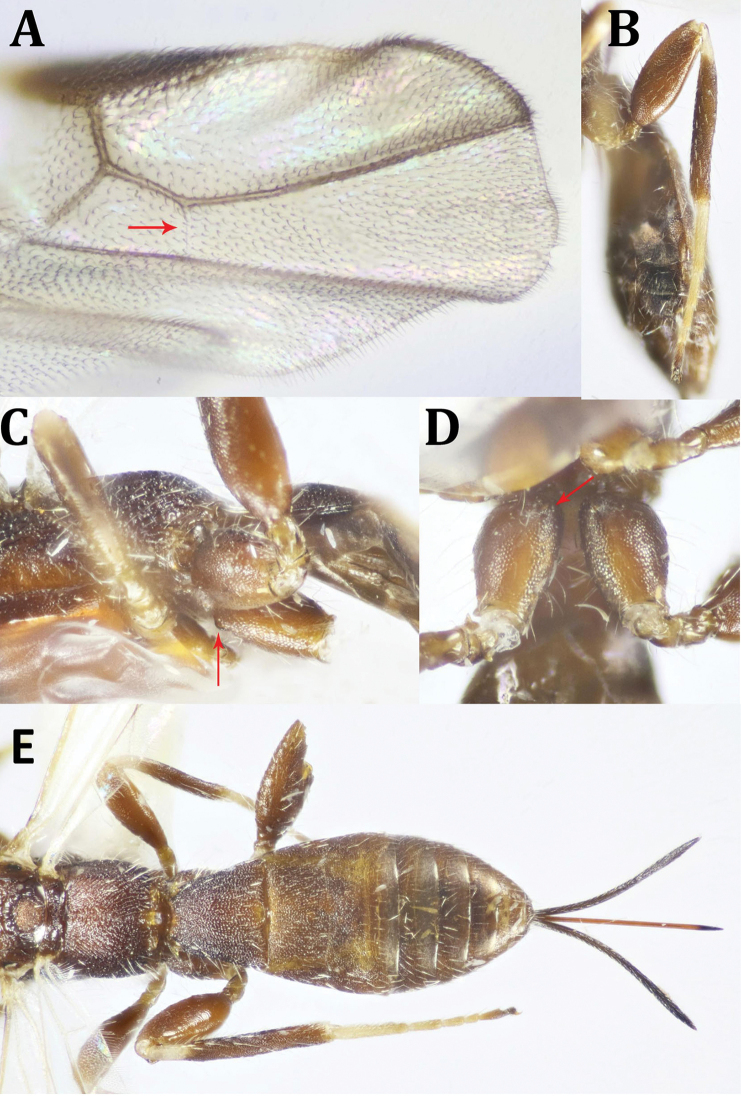
*Mimodoryctes arabicus* Edmardash, Gadallah & Soliman, sp. nov. ♀: **A** fore wing (part), presence of r-m indicated **B** hind leg and metasoma (part), lateral view **C** hind coxa, lateral view (basoventral tubercle indicated) **D** hind coxae, ventral view (basoventral tubercle indicated) **E** propodeum and metasoma, dorsal view.

Figures 12A–E , 13A–D , 14A–E

##### Type material.

Holotype: Kingdom of Saudi Arabia. ♀, Jazan, Farasan Islands, Al-Sajid; 16°51'25.46"N, 41°55'58.78"E; 10 Nov. 2017; Abu El-Ghiet & El-Sheikh leg.; LT [KSMA].

##### Description of holotype (female)

: Body length: 4.0 mm; ovipositor length: 1.0mm; fore wing length: 2.5 mm.

*Head* (Fig. 12C–E): 1.3× as wide as its median length, densely transversely striated in dorsal view; face coarsely rugose; frons not concave, without median carina, just a smooth slim area medially extending from between behind antennal bases, reaching clypeus; gena finely, obliquely striated; vertex and face sparsely setose. Temple roundly constricted behind eye, 0.5× as long as eye height. Clypeus coarsely rugose. Ocelli small; ocellar triangle with base 1.5× as long as its sides; POL 1.6× OD, 0.8× OOL. Eyes 2.1× as high as its width, with sparse short setae. Malar space 0.5× eye height, 0.6× basal width of mandible. Face width 0.9× eye height; hypostomal depression small, rounded, its width 0.9× distance of depression from eye edge. Head gently narrowly rounded behind eye when seen from frontal view. Antenna slender, broken (with 11 flagellomeres after being broken); scape short, 1.9× as long as its apical width; F1 slightly curved, 6.0× as long as its apical width, 1.1× as long as F2.

*Mesosoma* (Fig. 13A, B): 2.4× as long as its height. Mesoscutum not elevated above pronotum in lateral view. Pronotum with weak transverse carinae; mesoscutum flattened, coarsely rugose, with irregularly scattered fine setae, with a nearly smooth postero-medial area. Notauli indistinct. Mesoscutellum slightly convex to nearly flattened, ca. as long as its basal width, finely transversely puncticulate. Propodeum not areolate, with an incomplete median sulcus that is branched laterally giving off irregular oblique ridges. Mesopleuron coarsely rugose above, smooth with some fine punctures ventrally; sternaulus deep, nearly straight, extending along the entire ventral margin of mesopleuron.

*Wings* (Figs 13D, 14A): Fore wing 4.3× as long as its maximum width; metacarpus slightly longer than pterostigma (1.17×); pterostigma 4.7× as long as its maximum width; r arising from middle of pterostigma; 2-SR 1.5× r; 2-SR 0.3× SR1; m-cu distinctly antefurcal; vein 1cu-a postfurcal; distance between cu-a to 1-M 2.0× as long as cu-a; vein M+CU distinctly curved away from 1–1A; 1-CU1 0.4× 2-CU1; r-m not tubular, with wide bulla; 2-SR+M present. Hind wing with three hamuli on R1; vein SC+R 0.7× as long as vein C+SC+R; vein M+CU slightly longer than vein 1M (1.14×); vein m-cu interstitial, directed towards wing base.

*Legs* (Figs 13C, 14B–D): Hind coxa 2.4× as long as its maximum width, with a small rounded tubercle basoventrally, finely alutaceous, with a medio-ventral smooth and shiny area extending subbasally to apex; hind femur 2.6× as long as its maximum width, finely alutaceous, with some fine long hairs; outer edge of hind tibia with fine, long outstanding setae, ca. as long as tibial maximum width; hind tarsus ca. as long as hind tibia; hind basitarsus 0.7× as long as second-fifth tarsomeres combined.

*Metasoma* (Fig. 14E): slightly longer than head and mesosoma combined (1.1×). T1 distinctly gradually widened from base to apex, without spiracular protuberance, without basal carina; apical width of T1 3.0× its basal width, 1.2× as wide as its median length. T2 1.2× as wide as its middle length, with very weak median, slightly wavy, sulcus, 3.1× as long as T3. T1 and T2 (except posterior half of T2) densely granulose; posterior half of T2 and rest of tergites are smooth and shiny. Ovipositor distinctly shorter than metasoma, Ovipositor sheath 0.5× metasomal length, 1.7× T1 length.

*Color* (Figs 12A–C, E): Body dark reddish brown, with head and antennal flagellomeres lighter in color; palpi reddish brown. Legs dark reddish brown, except for pale yellow to ivory bases of tibiae and tarsi (except dark brown telotarsi). Wings hyaline, with slight infuscation under metacarpus as well as veins linings; veins dark brown with the following veins are pale: M+CU1 (except apically), 1-M, apical two-thirds of 2-CU1, m-cu. In hind wing, only 1r-m and distal half of 1-M are dark brown, rest of veins are pale.

##### Recognition.

The most important character separating the new species, *M. arabicus*, from the Algerian species *M. proprius* Belokobylskij is the presence of vein r-m of fore wing (Fig. 14A) (absent in *M. proprius*). Other characters can be summarized as follows: vertex transversely strigated without dense granulations between the striae (Fig. 12D) (in *M. proprius* dense granulations between striae could be seen); malar space relatively short, 0.6× basal width of mandible (Fig. 13A) (longer in *M. proprius*, 0.9× basal width of mandible); mesosoma 2.4× as long as high (Fig. 13A) (twice as long as high in *M. proprius*); propodeum with curved striations especially laterally (Fig. 13B) (densely striated in *M. proprius*); metasomal T1 and T2 densely rugulose-striated (Fig. 14E) (densely striated longitudinally in *M. proprius*); T4–6 finely sculptured at base (Fig. 14E) (in *M. proprius* the larger part of T3 with fine granulation, T4–6 with very weak granulation at base); body color dark reddish brown, including the legs except for bases of tibiae and all tarsi pale yellowish (Fig. 12A, B) (in *M. proprius*, body pale reddish brown, yellow in places, with the legs same as body with all tibiae yellowish at bases and apices); hind wing vein M+CU 1.2× 1-M (1.4× in *M. proprius*).

##### Remark.

The absence or presence of vein r-m of the fore wing has been found to be a polymorphic character for four genera: *Afrospathius* Belokobylskij & Quicke, *Leluthia* Cameron, *Pareucorystes* Tobias, and *Platydoryctes* Barbalho & Pentiado-Dias. However, this character has not yet been recorded in *Mimodoryctes* Belokobylskij (see Belokobylskij (2001)), and this was later confirmed in Belokobylskij et al. (2004) in their phylogenetic study of the doryctine genera based solely on morphological evidence. However, in the absence of other reliable diagnostic characters, the situation is considered in the present study to be the same as in the above-mentioned four genera.

#### Mimodoryctes proprius

Taxon classificationAnimaliaHymenopteraBraconidae

Belokobylskij, 2001

3B975717-90F5-5DCA-A923-03F44A823873

**Figure 15.**
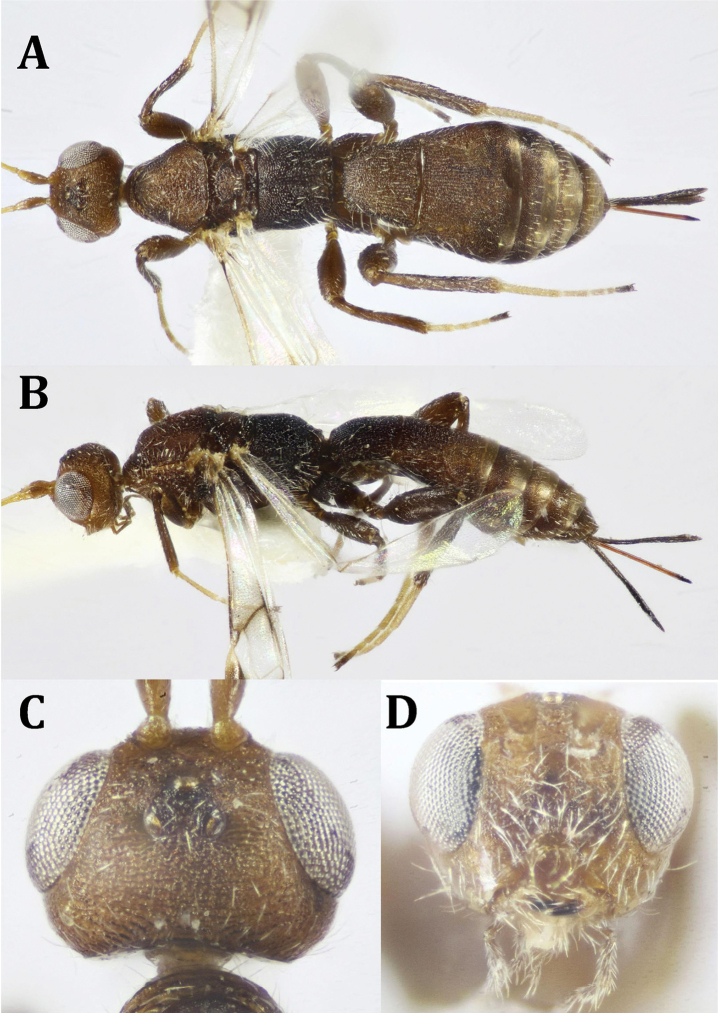
*Mimodoryctes proprius* Belokobylskij, ♀: **A** dorsal habitus **B** lateral habitus **C** head, dorsal view **D** head, frontal view.

**Figure 16.**
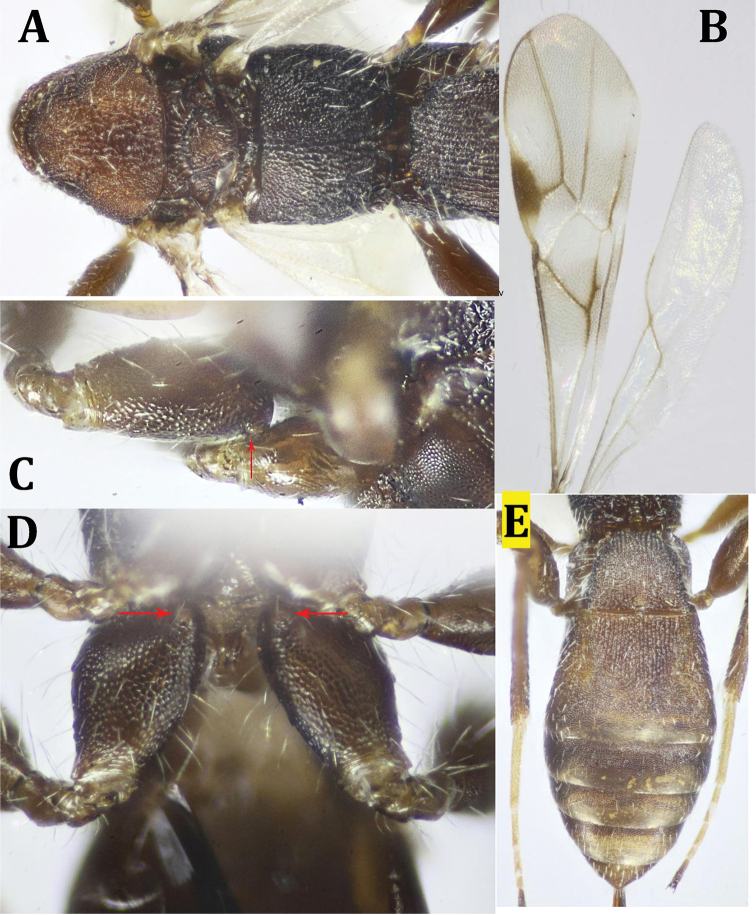
*Mimodoryctes proprius* Belokobylskij, ♀: **A** mesosoma and T1 (part) **B** fore and hind wings **C** hind coxa, lateral view (basoventral tubercle indicated) **D** hind coxae, ventral view (basoventral tubercle indicated) **E** metasoma, dorsal view.

Figures 15 (A–D), 16 (A–E)

Mimodoryctes proprius Belokobylskij, 2001: 750, ♀.

##### Re-description.

Body length: 3.6 mm; length of fore wing: 2.75 mm.

*Head* (Fig. 15C, D): 1.4× as wide as its median length, somewhat angulate behind eye in frontal view, roundly narrowed after eyes in dorsal view. Transverse eye diameter ca. twice as long as temple in dorsal view. Vertex with transverse curved striations with rugosity between striae (Fig. 15D). Face densely punctate, with fine, inwardly directed whitish setae, as well as thicker and shorter sparse setae on vertex. Temple gently rounded behind eyes, ca. 0.5× eye height. Ocelli small, ocellar triangle equilateral; POL 1.1× OD, 2.4× OOL. Eyes 1.2× as high as its width, glabrous. Malar space 0.4× as long as eye height, 1.6× as long as basal width of mandible. Face slightly wider than eye height (1.1×); hypostomal depression of moderate size, rounded, its width as long as its distance from eye edge. Antenna slender, with apex missing, 18-segmented, appearing shorter than body; scape 1.9× as long as its apical width, slightly longer dorsally than ventrally, F1 slightly curved along outer side, 6.3× as long as its apical width, slightly longer than F2 (1.2×); F3 straight, slightly longer than F4 (1.2×).

*Mesosoma* (Fig. 16A): 3.0× as long as its height. Mesoscutum gently and roundly elevated above pronotum. Pronotum with weak transverse carinae on the disc, without any processes, deeply concave posteriorly; mesoscutum flattened, sparsely setose, finely granulose anteriorly and laterally, coarsely rugose medially; notauli hardly seen; scuto-scutellar sulcus in the form of oval longitudinal depressions separated by carinae. Mesoscutellum ca. as long as its basal width, finely granulose on the disc, rugose laterally, sparsely setose apically. Propodeum without distinct areas, finely granulose at base, rest of it coarsely obliquely reticulate-rugose, sparsely setose laterally. Mesopleuron sparsely, superficially punctate above, finely granulose below, sternaulus superficially finely punctate, with row of 3–4 fine setae.

*Wings* (Fig. 16B). Fore wing 3.7× as long as its maximum width. Metacarpe 1.1× as long as pterostigma. Pterostigma 4.3× as long as its width; r released from the middle of pterostigma; 2-SR ca. as long as r; r-m absent, m-cu distinctly prefurcal; distance between cu-a to 1-M 0.1× cu-a length; 1-CU1 0.4× as long as 2-CU1; M+CU1 straight to slightly curved; 2-SR+M present, unsclerotized. Hind wing m-cu prefurcal.

*Legs* (Fig. 16C, D). Hind coxa 1.35× as long as its maximum width, densely alutaceous, with a small rounded tubercle basoventrally; hind femur 2.7× as long as its maximum width, finely alutaceous. Outer edge of hind tibia with a row of widely separated spines; hind tarsus slightly longer than hind tibia, 1.1×; hind basitarsus 0.8× as long as 2^nd^ -5^th^ tarsomeres combined.

*Metasoma* (Fig. 16E). 0.95× as long as head and mesosoma combined. T1 gradually widened from base to apex, 1.3× as wide as its middle length, without median longitudinal carina, with dense, close longitudinal striae, granulose in between; T2 distinctly broader than T1, 1.3× as wide its median length, longitudinally striated at anterior 0.7 length, followed by small, finely granulated area, then smooth at posterior margin, with very weak, hardly seen transverse curved sulcus medially; T1 0.7× as long as T2. Rest of tergites finely alutaceous, and smooth apically. Metasomal tergites sparsely setose. Ovipositor 0.4× as long as metasomal length, 1.8× as long as T1.

*Color* (Figs 15A, B, 16B). Body dark brown, with somewhat lighter head (face) and mesoscutum; eyes whitish. Legs dark brown, with yellowish tarsi (except dark brown to black telotarsi). Ovipositor red, black at tip; ovipositor sheath black. Fore wing with dark brown pterostigma, whitish at base; veins dark, with M+CU1 (except dark apically), 1-SR+M, m-cu and 2-SR+M, apical half of 2-CU1 membranous.

##### Material examined.

2♀, Kingdom of Saudi Arabia, Jazan, Farasan Islands, Al-Kosar; 16°40'5.75"N, 42°08'51.62"E, 25.I.2017; leg. Abu El-Ghiet & El-Sheikh; LT [KSMA].

##### Intraspecific variation.

The Saudi Arabian specimen differs from the Algerian one in the following: Vertex with transverse curved striation with rugosity between , frons and face coarsely rugose, weakly striated below eyes; temples weakly concentrically striated (vertex, frons densely striated, temple densely granulate); POL 1.6× OD, 0.95× OOL (1.3× OD, 0.75× in *proprius*); malar space 0.9× basal width of mandible (1.1× in *M. proprius*); ovipositor sheath 0.5× as long as metasomal length, 1.8× as long as T1 (0.35× metasomal length, 1.5× T1 in *M. proprius*).

##### General distribution.

Algeria (Belokobylskij, 2001), Saudi Arabia (Farasan Islands) (new record).

## Discussion

Saudi Arabia is a large arid land, covering the major part of the Arabian Peninsula, with an area of ca. 2,250,000 km^2^ (Aldhebiani and Howladar 2015). It is characterized by different ecosystems and is considered as one of the richest areas of biodiversity in the Arabian Peninsula, as its flora is formed by a mixture of Afrotropical, Oriental, and South Palaearctic (Mediterranean) elements (Aldhebiani and Howladae 2015).

From a biogeographical point of view, the position of Saudi Arabia is on the frontier between the Palaearctic and Afrotropical regions, as the Arabian Desert being a strong ecological barrier. The Farasan Archipelago (east of the Saudi Arabia-Yemen border) is considered to be more closely related to the Afrotropical region, with a high floristic diversity in relation to other parts of Saudi Arabia (Alwelaie et al. 1993; El-Demerdash 1996; Alfarhan et al. 2002).

In the Afrotropical region, the subfamily Doryctinae is represented by 234 species in 39 genera (Yu et al. 2016). Only three doryctine species are reported to occur in the Arabian Peninsula, *Rhaconotus arabicus*, *Zombrus anisopus* (Saudi Arabia) (Marshall 1900; Fahringer 1930; Fischer 1980; Belokobylskij 2001), and *Doryctophasmus ferrugineus* (United Arab Emirates, Yemen) (Belokobylskij 2015). In the present study, six doryctine species are added to the Arabian Peninsula fauna and Saudi Arabia (Farasan Archipelago), of which *Mimodoryctes arabicus* Edmardash, Gadallah & Soliman, and most probably *Neoheterospilus* sp. (until being confirmed by the collection of females) are new species. Most of the collected species are exclusively Afrotropical. This is closely correlated with the floristic composition of the area under study (Farasan Islands) as has been reported by many authors (e.g., Alwelaie et al. 1993; El-Demerdash 1996; Alfarhan et al. 2002).

In the present study, *Hecabalodes anthaxiae* Wilkinson, 1929 is recorded from Saudi Arabia, a species not recorded anywhere since it was originally described from Sudan (Wilkinson 1929).

The absence or presence of vein r-m of the fore wing has been found to be a polymorphic character for only four genera: *Afrospathius* Belokobylskij & Quicke, *Leluthia* Cameron, *Pareucorystes* Tobias, and *Platydoryctes* Barbalho & Pentiado-Dias. However, this character is absent in *Mimodoryctes* Belokobylskij (see Belokobylskij (2001)), and this was also confirmed in Belokobylskij et al. (2004) in their phylogenetic study of the doryctine genera based solely on morphological evidence. However, in the absence of other reliable diagnostic characters, the situation is considered in the present study to be the same as in the above-mentioned four genera. On the other hand, the number of segments in maxillary and labial palps can also be hardly counted especially in dry specimens, because the basal first and sometimes second segments can be very short and are very difficult to see separately in dry specimens (Belokobylskij, pers. comm.), and in our opinion, this character should also be considered as a polymorphic character for this genus.

Because of the rich biodiversity of Saudi Arabia, more species of this subfamily and others are expected to occur. Therefore, further collections and studies are needed to clarify the distribution of this group of wasps in other parts of this large country.

## Supplementary Material

XML Treatment for Dendrosotinus

XML Treatment for Dendrosotinus ferrugineus

XML Treatment for Hecabalodes

XML Treatment for Hecabalodes anthaxiae

XML Treatment for Neoheterospilus

XML Treatment for Neoheterospilus

XML Treatment for Rhaconotus

XML Treatment for Rhaconotus (Rhaconotus) carinatus

XML Treatment for Mimodoryctes

XML Treatment for Mimodoryctes arabicus

XML Treatment for Mimodoryctes proprius

## References

[B1] Aldhebiani AY Howladar SM (2015) Floristic diversity and environmental relations in two valleys, South West Saudi Arabia. International Journal of Science and Research 4(2): 1916–1925. https://www.ijsr.net/get_abstract.php?paper_id=SUB151587

[B2] Alfarhan AH Al Turki AT Thomas J Basahy RA (2002) Annotated list to the flora of Farasan Archipelago, Southern Red Sea, Saudi Arabia. Teckholmia 22(1): 1–33. 10.21608/taec.2002.12388

[B3] Alwelaie AN Chaudary SA Alwetaid Y (1993) Vegetation of some Red Sea islands of the Kingdom of Saudi Arabia. Journal of Arid Environment 24: 287–296. 10.1006/jare.1993.1025

[B4] Belokobylskij SA (1983) To the knowledge of the genera *Heterospilus* Hal. and *Dendrosotinus* Tel. (Hymenoptera, Braconidae) of the USSR fauna. Proceedings of the All-Union Entomological Society 65: 168–186.

[B5] Belokobylskij SA (1992) On the classification and phylogeny of the braconid wasps subfamilies Doryctinae and Exothecinae (Hymenoptera: Braconidae). I. Classification, 1. Entomologicheskoe Obozrenie 71: 900–928 (in Russian); Entomological Review 72: 109–137. https://ci.nii.ac.jp/naid/10011409279/

[B6] Belokobylskij SA (1994) The species of the genus *Heterospilus* Haliday, 1836 (Hymenoptera: Braconidae) from Vietnam. Tropical Zoology 7: 11–23. 10.1080/03946975.1994.10539237

[B7] Belokobylskij SA (1995) Principal evolutionary transformations of morphological structures in subfamilies Doryctinae and Exothecinae (Hymenoptera: Braconidae). Entomologicheskoe Obozrenie 74: 153–176. http://www.zin.ru/labs/insects/Hymenopt/Personalia/Belokobylskij/pdf/074-1995e.pdf

[B8] Belokobylskij SA (2001) New taxa of the braconid subfamilies Doryctinae and Exothecinae (Hymenoptera, Braconidae) from the Western Palaearctic region. Entomological Review 81(7): 749–766. http://www.zin.ru/labs/insects/Hymenopt/Personalia/Belokobylskij/pdf/112-2001e.pdf

[B9] Belokobylskij SA (2006) *Neoheterospilus* gen. n., a new genus of the tribe Heterospilini (Hymenoptera: Braconidae, Doryctinae) with highly modified ovipositor and a worldwide distribution. Insect Systematics and Evolution 37: 149–178. 10.1163/187631206788831119

[B10] Belokobylskij SA (2015) Review of species of the Old World genus *Doryctophasmus* Enderlein, 1912 (Hymenoptera: Braconidae: Doryctinae). Zootaxa 3985(4): 541–564. 10.11646/zootaxa.3985.4.4 26250163

[B11] Belokobylskij SA Maeto K (2008) Doryctinae (Hymenoptera: Braconidae) of Ogasawara Islands (Japan). Annales Zoologici (Warszawa) 58(1): 125–166. 10.3161/067.058.0107

[B12] Belokobylskij SA Zaldívar-Riverón A Quicke DLJ (2004) Phylogeny of the genera of the parasitic wasps, subfamily Doryctinae (Hymenoptera: Braconidae) based on morphological evidence. Zoological Journal of the Linnean Society 142: 369–404. 10.1111/j.1096-3642.2004.00133.x

[B13] Braet Y (2016) Key to the Genera of Doryctinae of the World. http://www.doryctinaekey.myspecies.info

[B14] Cameron P (1910) On some Asiatic species of the subfamilies Spathiinae, Doryctinae, Rhogadinae, Cardiochilinae and Macrocentrinae in the Royal Berlin Zoological Museum. Wiener Entomologische Zeitschrift 29: 93–100. 10.5962/bhl.part.23337

[B15] Chen XX van Achterberg C (2019) Systematics, phylogeny, and evolution of the braconid wasps: 30 years of progress. Annual Review of Entomology 64: 1–24. 10.1146/annurev-ento-011118-111856 30332295

[B16] de Macêdo MV Monteiro RT (1989) Seed predation by a braconid wasp, *Allorhogas* sp. (Hymenoptera). Journal of the New York Entomological Society 97: 358–362. https://www.jstor.org/stable/25009780

[B17] Dowton M Austin AD Antolin MF (1998) Evolutionary relationships among the Braconidae (Hymenoptera: Ichneumonoidea) inferred from partial 16S rDNA gene sequences. Insect Molecular Biology 7: 129–150. 10.1046/j.1365-2583.1998.72058.x 9535159

[B18] El-Demerdash MA (1996) The vegetation of the Farasan Islands, Red Sea, Saudi Arabia. Journal of the Vegetation Science 7: 81–88. 10.2307/3236419

[B19] Fahringer J (1930) Opuscula braconologica. Band 3. Palaearktischen Region. Liefrung 1–2. Opuscula braconologica, 1–160.

[B20] Fischer M (1968) Über gezüchtete Raupenwespen (Hymenoptera: Braconidae). Pflanzenschutz Berichte 37(7/8/9): 97–140.

[B21] Fischer M (1980) Taxonomische Untersuchungen über Doryctinae aus der *Odontobracon* Verwandtschaft (Hymenoptera, Braconidae). Annalen des Naturhistorischen Museums in Wien 83: 547–572. https://www.jstor.org/stable/41768825

[B22] Foerster A (1863) Synopsis der Familien und Gattungen der Braconiden. Verhandlingen des Naturhistorischen Vereines der Pruessischen Rheinlande und Westphalens 19: 226–288.

[B23] Harris RA (1979) A glossary of surface sculpturing. Occasional Papers in Entomology 28: 1–31. http://agris.fao.org/agris-search/search.do?recordID=US201300583954

[B24] Hedqvist KJ (1965) Braconidae from the Cape Verde Islands. Commentationes Biologicae, Helsinki 28: 1–28.

[B25] Giraud J (1869) Observations hyménoptèrologiques. Annales de la Société Entomologique de France (4) 9: 469–488.

[B26] Mandura AS Saifullah SM Khafaji AK (1987) Mangrove ecosystem of southern Red Sea Coast of Saudi Arabia. Proceedings of Saudi Biological Society 10: 165–193.

[B27] Marsh PM (1991) Description of a phytophagous doryctine braconid from Brazil (Hymenoptera: Braconidae: Doryctinae). Proceedings of the Entomological Society of Washington 93(1): 92–95. https://www.cabdirect.org/cabdirect/abstract/19921158253

[B28] Marsh PM (1993) Descriptions of new Western Hemisphere genera of the subfamily Doryctinae (Hymenoptera: Braconidae). Contributions of the American Entomological Institute 28(1): 1–58. http://www.sidalc.net/cgi-bin/wxis.exe/?IsisScript=oet.xis&method=post&formato=2&cantidad=1&expresion=mfn=026854

[B29] Marsh PM (1997) Doryctinae. In: Wharton RA Marsh PM Sharkey MJ (Eds) Manual of the New World Genera of the Family Braconidae (Hymenoptera).International Society of Hymenopterists. Special Publication No.1, 207–233.

[B30] Marsh PM (2002) The Doryctinae of Costa Rica (excluding the genus *Heterospilus*). Memoirs of the American Entomological Institute 70: 1–319. http://www.sidalc.net/cgibin/wxis.exe/?IsisScript=oet.xis&method=post&formato=2&cantidad=1&expresion=mfn=024700

[B31] Marsh PM de Macêdo MV Pimental MCP (2000) Descriptions and biological notes on two new phytophagous species of the genus *Allorhogas* from Brazil (Hymenoptera: Braconidae: Doryctinae). Journal of the Hymenoptera Research 9(2): 292–297. https://www.biodiversitylibrary.org/page/2858335

[B32] Marsh PM Wild AL Whitfield JB (2013) The Doryctinae (Braconidae) of Costa Rica: genera and species of the tribe Heterospilini. ZooKeys 347: 1–474. https://www.ncbi.nlm.nih.gov/pmc/articles/PMC3822444/10.3897/zookeys.347.6002 PMC3822444 24222723

[B33] Marshall TA (1888) Les Braconides. In: André E (Ed.) Species des Hyménoptères d’Europe et d’Algérie. Beaune and Gray, Paris, 4, 609 pp.

[B34] Marshall TA (1900) Les Braconides (Supplément). In: André E (Ed.) Species des Hyménoptères d’Europe et d’Algerie. Paris-Tome 5 bis, 369 pp.

[B35] Muoftah IA (1990) Farasan: People, Sea and History. Jizan Cultural Club, Jizan.

[B36] Papp J (1987) Braconidae (Hymenoptera) from Korea. VIII. Acta Zoologica Hungarica 33: 157–175. https://pascalfrancis.inist.fr/vibad/index.php?action=getRecordDetail&idt=8333549

[B37] Polaszek A Fitton MG Bianchi G Huddleston T (1994) The parasitoids of the African white rice borer, *Maliarpha separatelle* Ragonot (Lepidoptera: Pyralidae). Bulletin of the Entomological Research 84: 65–90. 10.1017/S0007485300032247

[B38] Quicke DLJ (2015) The Braconid and Ichneumonid Parasitoid Wasps: Biology, Systematics, Evolution and Ecology. Hoboken, N.J. Wiley Blackwell, 681 pp 10.1002/9781118907085

[B39] Quicke DLJ Ficken LC Fitton MG (1993) New diagnostic ovipositor characters for doryctine wasps (Hymenoptera: Braconidae). Journal of the Natural History 26: 1035–1046. 10.1080/00222939200770611

[B40] Ramírez WB Marsh PM (1996) A review of the genus *Psenobolus* (Hymenoptera: Braconidae) from Costa Rica, an inquiline fig wasp with brachypterous males, with description of two new species. Journal of the Hymenoptera Research 5: 64–72. http://repositorio.sibdi.ucr.ac.cr:8080/jspui/bitstream/123456789/11873/1/RBW0004.pdf

[B41] Ruthe JF (1854) Beiträge zur Kenntnis der Braconiden. Stettiner Entomologische Zeitung 15: 343–355.

[B42] Sharanowski BJ Dowling APG Sharkey MJ (2011) Molecular phylogenetics of Braconidae (Hymenoptera: Ichneumonoidea), based on multiple nuclear genes, and implications for classification. Systematic Entomology 36: 549–572. 10.1111/j.1365-3113.2011.00580.x

[B43] Sharkey MJ Wharton RA (1997) Morphology and terminology. In: Wharton RA Marsh PM Sharkey MJ (Eds) Manual of the New World Genera of the Family Braconidae (Hymenoptera).International Society of Hymenopterists. Special Publication No.1, 19–63.

[B44] Shaw MR (1995) Observations on the adult behaviour and biology of *Histeromerus mystacinus* Wesmael (Hymenoptera: Braconidae). Entomologist 114(1): 1–13.

[B45] Shaw MR (1997) The genus *Heterospilus* Haliday in Britain with description of a new species and remarks on related taxa (Hymenoptera: Braconidae: Doryctinae). Zoologische Mededelingen Leiden 71(5): 33–41. http://repository.naturalis.nl/document/149389

[B46] Shaw SR Edgerly JS (1985) A new braconid genus (Hymenoptera) parasitizing web-spinners (Embiidina) in Trinidad. Psyche 92: 505–511. 10.1155/1985/54285

[B47] Shenefelt RD Marsh PM (1976) Pars 13. Braconidae 9, Doryctinae. In: Vecht der Van Shenefelt RD (Eds) Hymenopterorum Catalogus (nova editio).Dr W. Junk, The Hague, 1263–1424. https://ci.nii.ac.jp/naid/10011409284/

[B48] Shi QX Yang JQ Chen JH (2002) A new species of *Heterospilus* Haliday (Hymenoptera: Braconidae) from China. Entomological Journal of East China 11(2): 3–5. https://europepmc.org/article/cba/383682

[B49] Strumia F Dawah H (2019) An overview of the Chrysididae (Hymenoptera) of the Red Sea Farasan Archepelago (Saudi Arabia). Journal of the Insect Biodiversity 9(1): 1–17. 10.12976/jib/2019.09.1.1

[B50] Tang P Belokobylskij SA He JH Chen XX (2013) *Heterospilus* Haliday (Hymenoptera: Braconidae, Doryctinae) from China with a key to species. Zootaxa 3683(3): 201–246. 10.11646/zootaxa.3683.3.1 25250449

[B51] Telenga NA (1941) Family Braconidae, subfamily Braconinae (continuation) and Sigalphinae. Fauna USSR. Hymenoptera 5(3): 1–466.

[B52] Tobias VI Belokobylskij SA Kotenko AG (1995) Keys to the insects of the European part of the USSR. Akademia Nauk SSSR, Zoologicheskii Institut, 908 pp https://www.cabdirect.org/cabdirect/abstract/19961104913

[B53] van Achterberg C (1993) Illustrated key to the subfamilies of the Braconidae (Hymenoptera: Ichneumonoidea). Zoologische Verhandelingen 283: 1–189. https://ci.nii.ac.jp/naid/10008879101/

[B54] van Achterberg C Polaszek A (1996) The parasites of cereal stem borers (Lepidoptera: Cossidae, Crambidae, Noctuidae, Pyralidae) in Africa, belonging to the family Braconidae (Hymenoptera: Ichneumonoidea). Zoologische Verhandelingen 204: 1–123. https://ci.nii.ac.jp/naid/10008879101/

[B55] van Achterberg C Walker AK (1998) 17 Braconidae In: Polaszek A (Ed.) African Stem Borers – Economic importance, taxonomy, natural enemies and control.CAB International, Wallingford, 137–185, 448–483. http://www.sidalc.net/cgibin/wxis.exe/?IsisScript=UACHBC.xis&method=post&formato=2&cantidad=1&expresion=mfn=091907

[B56] van Achterberg C Marsh PM (2002) Revision of the genus *Psenobolus* Reinhard (Hymenoptera: Braconidae: Doryctinae). Zoologische Mededelingen Leiden 76(1): 1–25. https://repository.naturalis.nl/pub/217457/ZM76_001-026.pdf

[B57] Wilkinson DS (1929) New parasitic Hymenoptera and notes on other species. Bulletin of Entomological Research 20(1): 103–114. 10.1017/S000748530002099X

[B58] Yu DSK van Achterberg C Horstmann K (2016) Taxapad 2016, Ichneumonoidea 2015. Taxapad database on flash-drive, Ottawa.

[B59] Zaldívar-Riverón A Mori M Quicke DLJ (2006) Systematics of the cyclostome subfamilies of braconid parasitic wasps (Hymenoptera: Ichneumonoidea): A simultaneous molecular and morphological Bayesian approach. Molecular Phylogenetics and Evolution 38(1): 130–145. 10.1016/j.ympev.2005.08.006 16213168

[B60] Zaldívar-Riverón A Belokobylskij SA Leon RV Martínez J Brinceno R Quicke DLJ (2007) A single origin of gall association in a group of parasitic wasps with disparate morphologies. Molecular Phylogenetics and Evolution 44(3): 981–992. 10.1016/j.ympev.2007.05.016 17625923

[B61] Zaldívar-Riverón A Belokobylskij SA Leon RV Martínez J Brinceno R Quicke DLJ (2008) Molecular phylogeny and historical biogeography of the cosmopolitan parasitic wasp subfamily Doryctinae (Hymenoptera: Braconidae). Invertebrate Systematics 22(3): 345–363. 10.1071/IS07028

[B62] Zaldívar-Riverón A Martínez J Belokobylskij SA Pedraza-Lara C Shaw SR Hanson PE Varela-Hernandez F (2014) Systematics and evolution of gall formation in the plant-associated genera of the wasp subfamily Doryctinae (Hymenoptera: Braconidae). Systematic Entomology 39(4): 633–659. 10.1111/syen.12078

